# Cardiomyocyte differentiation of mesenchymal stem cells from bone marrow: new regulators and its implications

**DOI:** 10.1186/s13287-018-0773-9

**Published:** 2018-02-26

**Authors:** Xiaofei Guo, Yan Bai, Li Zhang, Bo Zhang, Naufal Zagidullin, Katherine Carvalho, Zhimin Du, Benzhi Cai

**Affiliations:** 1 Department of Pharmacy, the Second Affiliated Hospital of Harbin Medical University, No. 246 Xuefu Road, Harbin, Heilongjiang Province 150081 People’s Republic of China; 2 Department of Internal Diseases, Bashkir State Medical University, Ufa, Russia; 3 Cell Therapy and Biotechnology in Regenerative Medicine Research Group, Pequeno Príncipe Faculty, Pelé Pequeno Príncipe Institute, Curitiba, Brazil

**Keywords:** Bone marrow-derived mesenchymal stem cells, Cardiomyocytes, Differentiation, microRNAs, Cytokines, Microenvironment

## Abstract

In the past years, cardiac mortality has decreased, but cardiac diseases are still responsible for millions of deaths every year worldwide. Bone-marrow mesenchymal stem cells (BMSCs) transplantation may be a promising therapeutic strategy because of its capacity to differentiate into cardiac cells. Current research indicates that chemical substances, microRNAs, and cytokines have biological functions that regulate the cardiomyocytes differentiation of BMSCs. In this review, we chiefly summarize the regulatory factors that induce BMSCs to differentiate into cardiomyocytes.

## Background

Cardiac diseases remain the leading cause of death worldwide, both in developed and developing countries. Cardiac diseases can progress rapidly, such as acute myocardial infarction (AMI), or progress slowly, such as cardiac remodeling, which is characterized by cardiac hypertrophy and myocardial fibrosis that can eventually lead to heart failure. Although, a variety of measures have been put into clinical practice and achieved certain curative effects, the poor prognosis and irreversible pathology of cardiac remodeling still limit their therapeutic effect for cardiac diseases.

Nowadays, significant advances have been made in the field of cardiac diseases and stem cell transplantation-based therapies have emerged as a promising therapeutic tool for improving cardiac regeneration and function [1–3]. In addition, pluripotent stem cells, including embryonic stem (ES) cells, induced pluripotent stem (iPS) cells, and multipotent/unipotent stem cells like bone marrow-derived mesenchymal stem cells (BMSCs) can be differentiated into cardiomyocytes in vitro [4–7]. However, the ideal source of stem cells remains elusive, with the drawbacks of limited engraftments and differentiation potential, ethical issues, and immunologic incompatibility. Of these stem cell types, BMSCs have several advantages of easy availability, powerful capacity of proliferation, immune modulatory properties, and migration to damaged tissues [8]. BMSC transplantation is considered a promising cardiac disease strategy due to differentiation [9–11]. Many efforts have been proved to facilitate differentiation of BMSCs into cardiomyocytes, such as chemical substances, microRNAs, and cytokines, or alter culture intermediaries [12–14].

## Chemicals

5-Azacytidine (5-aza) is an important chemical inducer that can induce BMSCs to differentiate into cardiomyocytes in murine samples [15]. Antonitsis et al. also found that 5-aza could stimulate BMSCs to differentiate into cardiomyocytes via random demethylation of DNA in the human body [16, 17]. Although 5-aza has been extensively used in stem cell differentiation, the carcinogenicity of 5-aza still blocks therapeutic applications [18] so other alternative options for BMSCs to differentiate into cardiomyocytes are imperative.

## MicroRNAs

MicroRNAs (miRNAs) are a class of noncoding RNAs about 22 nucleotides long that can act as negative regulators of gene expression by binding to the 3′ UTR of mRNAs [19]. Previous studies have confirmed that miRNAs can play a significant role in cell development, differentiation, proliferation, and apoptosis [20]. Studies have also demonstrated that miRNAs can regulate differentiation of BMSCs [21, 22]. However, whether miRNAs could regulate cardiomyocyte differentiation of BMSCs is still little known.

Zhao et al.’s [23] study reveals that miR-1a could promote the differentiation of BMSCs into myocardial cells. Their results show that BMSCs could differentiate into myocardial cells in special conditioned medium, but will be efficient when overexpressing miR-1a. As an in-depth study, they demonstrate that Delta-like 1 (Dll-1) is the key inhibitor of myocardium gene expression during myocardium differentiation and that miR-1a can reduce the expression of Dll-1 by targeting the 3′ UTR, leading to the dramatic upregulation of myocardium gene protein.

Cai et al. [24] show that BMSCs are transformed into cardiomyocytes by coculture with cardiomyocytes, and cardiac-specific markers such as atrial natriuretic peptide (ANP), cardiac troponin T (cTnT), and α-myosin heavy chain (α-MHC) are detected. miRNA assay indicates that the level of miR-124 is significantly downregulated during cardiomyocte differentiation of BMSCs. The authors then performed functional experiments on the acquisition or loss of miR-124 and find that overexpression of miR-124 would inhibit cardiomyocyte differentiation of BMSCs. By further study into the molecular mechanism of this progress, the authors demonstrate that miR-124 exerts a negative effect on myogenic differentiation of BMSCs via targeting signal transducers and activators of transcription 3 (STAT3) [24].

Shen et al. [25] find the expression of miR1-2 is significantly increased after 5-aza treatment. In order to clear the role of miR1-2 in modulating cardiomyocyte differentiation, miR1-2 mimics are transferred into BMSCs, and these cells are induced to differentiate into cardiomyocytes by the expression of cardiac-specific genes GATA binding protein 4 (GATA4), cardiac troponin I (cTnI), and Homeobox protein 2.5 (Nkx2.5). Further study shows that miR1-2 could activate the Wnt/β-catenin signaling pathway, whereas BMSCs pretreated with Wnt/β-catenin signaling inhibitor LGK-974 can weaken the differentiation of BMSCs into cardiomyocytes. To sum up, miR1-2 could regulate the differentiation of BMSCs into cardiomyocytes via the Wnt/β-catenin signaling pathway [25].

miRNAs could regulate gene expression and the cardiomyocyte development and differentiation of stem cells [26], including BMSCs. For instance, miR-23b inhibits the osteogenic differentiation of BMSCs via targeting Runx2 during treatment with TNF-α [27]. The miR-1/133 family has a high level in the heart, but they have opposing effects: miR-1 promotes and miR-133 blocks differentiation into cardiac cells. A previous study has shown that Jagged 1 protein could activate Notch signal and promote the differentiation of BMSCs into cardiomyocytes in vitro and in vivo [28], and miR-1 could promote myocardial differentiation in stem cells via targeting Dll-1, a Notch ligand expressed in ES cells [29]. The Wnt signaling pathway has an essential role in cardiomyocyte development and β-catenin could promote the occurrence of the heart in Drosophila [30]. The Wnt signaling pathway also regulates the proliferation and differentiation of BMSCs [31]. miR-29c-3p is significantly upregulated and could regulate the osteoblast differentiation of rat BMSCs by targeting Dishevelled 2, a key mediator of the Wnt/β-catenin signaling pathway, in a hyperlipidemia environment [32]. Therefore, the increasing in miR-124 level is considered to be an important trigger of the transition from proliferation to neural differentiation [33]. STAT3 has a significant role in self-renewal, differentiation, and paracrine activation of BMSCs [7, 34]. Activation of STAT3 has been reported that could enhance the differentiation of transplanted BMSCs and produce better function of infarcted myocardium. miR-124 regulates the activation of STAT3 and in turn affects myogenic differentiation of BMSCs.

The miRNAs mimics group have a lower apopotic rate than the 5-aza group, indicating miRNAs are less cytotoxic. BMSCs treated with miRNAs express cardiac-specific genes but these cells are still short of the morphology of cardiomyocytes, indicating that further investigation needs to be done. Long noncoding RNAs have been shown to play important roles in multiple physiological processes. Nowadays, lncRNA H19 could mediate osteogenesis differentiation of BMSCs by sponging miR-138 [35]. This might be a new strategy to induce cardiomyocyte differentiation of BMSCs through miRNAs.

## Cytokines

### Growth factor

Growth factors such as epidermal growth factor, platelet-derived growth factor, fibroblast growth factor, nerve growth factor, and insulin-like growth factor are cytokines that can affect cell growth and differentiation. Whether it can regulate the differentiation of cardiomyocytes in BMSCs needs to be further explored.

#### Insulin-like growth factor-1

Insulin-like growth factor-1 (IGF-1) plays an important role in the regulation of cell proliferation, apoptosis, and tumorigenicity [36–38]. Growing evidence indicates that BMSCs have the potential to differentiate into cardiomyocyte-like cells (CLCs) which have the capacity for contractility and can express the cardiac-specific gene [39–41]. Whether IGF-1 could participate in cardiomyocytes differentiation of BMSCS, more experiments need to be done.

Transplanted BMSCs need a feasible microenvironment to differentiate into cardiomyocytes in the ischemic area [42]. In-vitro experiments suggest that HGF has a significant role in promoting myocardial differentiation of BMSCs, but lacking the ability to proliferate and inhibit apotpsis. However, IGF-1 could significantly supply the gap of HGF. Research has tried to investigate the effect of combination of two factors in AMI therapy [43], and the combination of IGF-1 and HGF could promote the cardioprotective effects of adipose-derived stem cells [44]. Zhang et al. [45] find that a combination of HGF and IGF-1 could inhibit BMSC apoptosis and increase angiogenesis. To further investigate whether HGF and IGF-1 could induce the cardiomyocyte differentiation of BMSCs, immunofluorescence staining, qRT-PCR, and western blot analysis are executed. After treatment of BMSCs with HGF and IGF-1, the level of cardiac-specific markers like cardiac troponin T (cTnT), GATA4, NKx2.5, and Connexin 43 (CX43) are all increased, suggesting that the combination of HGF and IGF-1 could achieve the dual purpose of not only promoting differentiation of BMSCs into cardiomyocytes but also inhibiting apoptosis induced by hypoxia [45]. However, the mechanism is not clear.

IGF-1 can affect cell proliferation, apoptosis, angiogenesis, and cardiac protection, especially the differentiation of stem cells, and a previous study shows that insulin-like growth factor could promote differentiation after transplanting ES cells for myocardial renovation [46]. IGF-1R is the significant individual component, and IGF/IGF-1R can be activated to enhance proliferation and survival of many cells [47]. Gong et al. [48] notice the increased expression of myocardium markers cTnT, cTnI, and phosphorylation IGF-1 receptor (pIGF-1R) after exposing BMSCs to IGF-1. To confirm whether the differentiation of BMSCs into CLCs is induced by IGF-1, BMSCs are treated with IGF-1 and I-OMe AG538, an IGF-1R kinase inhibitor that can block its autophosphorylation. The results show that the expression of cTnT and cTnI is decreased through the MAPK and PI3K pathways, which are the two major IGF-1R-related intracellular signaling pathways. In conclusion, IGF-1 induces the BMSCs to differentiate into CLCs via IGF-1R [48]. PI3K/Akt is the downstream signaling pathway of IGF-1, and plays a role in cell survival and migration [49], and the MAPK/ERK pathway could modulate the expression of proteins involved in differentiation. Whether the MAPK/ERK pathway participates in the processes of differentiating BMSCs into CLCs needs further research.

#### Insulin gene enhancer binding protein ISL-1

Insulin gene enhancer binding protein ISL-1 (Islet-1) is a significant regulator of cardiac development and cardiomyocyte differentiation [50, 51], and is also a marker of undifferentiated cardiac progenitors [52]. A previous study verifies that overexpressed Islet-1 in BMSCs could play a critical role in cardiomyocyte differentiation [52]. This is discussed in the context of how Islet-1 carries the differentiation function. Yi et al. [53] find that increasing expression of Islet-1 by lentiviral vector could promote the differentiation of MSCs into CLCs, and the level of GATA4 is also elevated. Further studies demonstrate that Islet-1 alters the histone acetylation levels of GATA4 and the DNA methylation levels of GATA4 promoter region through Gcn5 and DNMT-1 [53].

Epigenetic modifications, including histone acetylation and DNA methylation, have been demonstrated to serve an important role in cardiomyocyte differentiation of MSCs [54]. Histone acetylation changes the transcriptional activity of chromatin [55], while DNA methylation alters the function of DNA by methylation modifications [56]. Gcn5, the first discovered histone acetyltransferase, mainly modifies nucleosomal histones and free histones [57]. During the process of Islet-1-induced BMSC differentiation into CLCs, the expression of Gcn5 and binding to GATA4 promoter regions are both increased, subsequently enhancing the expression of GATA4 to promote the cardimoycyte differentiation of BMSCs. The main role of DNMT1 is to form DNA methylation, the expression of DNMT1 and binding to GATA4 promoter regions are both decreased in the process of Islet-1 induction. The function of two epigenetic modifications presents a crosscurrent and may regulate the expression of GATA4 reciprocally.

#### Basic fibroblast growth factor

Basic fibroblast growth factor (bFGF), one of the heparin binding growth factors, is thought to induce the differentiation of BMSCs into cardiomyocytes during embryogenesis [58], but not cardiomyocyte development [59], indicating that it is necessary for bFGF to combine with other factors in inducing cardiomyocyte differentiation of BMSCs. MSCs isolated from sternal marrow show potential to differentiate into mesodermal lineages, like osteocytes, adipocytes, and chondrocytes, and whether the MSCs have the potential to differentiate into cardiomyocytes is an interest question. Hafez et al. [60] find that bFGF synergizes with hydrocortisone, a steroid drug produced by the adrenal gland, to induce cardiomyocyte differentiation of sternal marrow MSCs. Immunofluorescence analysis and qRT-PCR show that cardiac markers like cTnI, cardiac troponin C (cTnC), and cardiac intracellular gap junction protein CX43 in bFGF and hydrocortisone-treated BMSCs are upregulated compared with 5-aza-treated BMSCs. These data suggest that the combination of bFGF and hydrocortisone can not only induce BMSCs to differentiate into cardiomyocytes, but is more efficient than treatment with 5-aza.

A previous study shows that bFGF could increase migratory activity, engraftment, and therapeutic potency [61]. bFGF could promote new arteriolar formation and LV functional improvements, and is essential in MSC angiogenesis and improving the ischemic surroundings [62, 63]. It also enhances differentiation of BMSCs for cardiac repair after myocardial infarction [64], suggesting that bFGF is important in cardiac diseases. Hydrocortisone has an important role in the regulation of cardiomyocyte proliferation and differentiation [60]. In the role of maintenance and differentiation of mesodermal cells, bFGF and hydrocortisone may be a good selection to induce cardiomyocyte differentiation. Experiments such as Hafe et al also prove this character [60].

### Interleukin

Cytokines could induce MSCs differentiating into cardiac lineage in the microenvironment. These factors can be derived by autocrine and paracrine signaling. Interleukin, a cytokine, is also vital for regulating cardiac function, development, and pathogenesis. The interleukin-1 family is a member of proinflammatory cytokines and has been divided into two types: IL-1α and IL-1β, which have biological function in a variety of cells, especially cardiomyocytes [65]. Recent studies have shown that IL-1β plays a critical role in pathogenesis, development, and function of cardiomyocytes in impaired heart [66]. A previous study demonstrates that IL-1β could mediate the neovascularization after myocardial ischemia [67] and cardiac development processes.

Khajeniazi [68] finds that IL-1β has a positive effect on cardiac differentiation as well as 5-aza in vitro. On pretreatment of BMSCs with IL-1β, myocardial marker proteins cTnI, cTnT, CX43, and α-cardiac actin are expressed in the process of differentiation. Then, BMSCs are treated with IL-1β and 5-aza, and the data show that the combination of IL-1β and 5-aza is more effective than using IL-1β or 5-aza respectively.

These results show that IL-1β has a potential role in promoting cardiomyocyte differentiation of BMSCs. The expression of Notch ligand Jagged1 can be induced by IL-1β on human dystrophic myogenic cells, which could promote cardiomyocyte differentiation [69]. Based on its pleiotropic features and these data, further studies are needed to delineate whether IL-1β could affect the differentiation process of MSCs into cardiomyocytes. Khajeniazi’s [68] result shows that IL-1β could induce differentiation of BMSCs into cardiomyocytes, similar to 5-aza, but when IL-1β is applied in combination with 5-aza they exert a synergistic impact on cardiomyocyte differentiation. However, the mechanism of IL-1β-induced cardiomyocyte differentiation is not clear. Wnt signaling pathway is essential in cardiomyocyte development and it has also been noted that IL-1β has the ability to induce osteogenic differentiation of hMSCs via Wnt-5a/receptor tyrosine kinase-like orphan receptor 2 pathways [70]. It can be suspected that IL-1β might come into play via the Wnt signaling pathway.

### TGF-β family

#### Transforming growth factor β1

TGF-β1 belongs to the TGF-β family, which can regulate a load of biological processes, including proliferation, survival, differentiation, and migration of various cells [71, 72]. After treatment with TGF-β1, murine BMSCs increase the expression of cardiac-specific markers, such as cTnI, cTnT, α-MHC, and α-sarcomeric actin, suggesting that TGF-β1 may promote differentiation of BMSCs into cardiomocytes [73]. Further studies report that BMSCs treated with autologous serum and TGF-β1 are significantly more sensitive than BMSCs treated with medium supplement containing 10% FBS and serum-free medium by detecting the expression of cTnT and GATA4, exhibiting a higher rate of proliferation and the capacity to differentiate into cardiomyocytes [74]. Electrical stimulation also affects the cardiomyocyte microenvironment [75], and would regulate the cariogenic differentiation of stem cells. Previous studies have found that electrical stimulation promotes the mRNA expression of GATA4 and Nkx2.5 in BMSCs [76], indicating that electrical stimulation could induce cardiomyocyte differentiation of BMSCs. The current study shows TGF-β1 may be involved in this process [77]. The expression of TGF-β1 is increased during electrical stimulation. To investigate whether electrical stimulation induces the cardiomyocyte differentiation of BMSCs through TGF-β1, BMSCs are treated with PFD (a TGF-β1 inhibitor); protein levels of the cardiac markers CX43 and α-actinin 2 are higher in the electrical stimulation group than in the electrical stimulation + PFD group, indicating that electrical stimulation induces the cardiomyocyte differentiation of BMSCs through TGF-β1.

TGF-β1 has known to be an important factor in embryonic heart development [78] and could induce cardiomyocyte differentiation of ES cells [79]. TGF-β1 also could promote the cardiomyocyte differentiation of skeletal muscle-derived adult primitive cells and the cardiomyocyte-like differentiation of BMSCs [74]. This result shows that TGF-β1 could induce BMSC cardiomyocyte differentiation. As the expansion of MSCs performed in cell culture medium with FBS has several problems including viral, bacterial, and prion [80], autologous serum has been considered. Autologous serum could regulate the proliferation and differentiation of MSCs [81] and enhance the cell viability. Chachques et al. [82] had transplanted autologous myoblasts which were cultured in autologous serum medium into LV infarcted patients to repair the jeopardized myocardium.

The electrical microenvironment is a key regulating factor of cardiomyocytes in vivo, and could trigger the cardiac-specific marker expression of various types of cells including fibroblasts [83], human mesenchymal stem cells [84], and ES cells [85]. Because of cardiac development of TGF-β1 during embryogenesis [86] and various cardiac pathologies [87], a hypothesis that whether electrical stimulation could increase the cardiomyocytes differentiation of BMSCs with TGF-β1 supplement is provided. The experiment demonstrates that electrostimulation would induce the cardiomyocyte differentiation of BMSCs via TGF-β1 with a higher efficiency. However, the exact molecular mechanism and the signaling pathway mediating this process remain unknown, making it clear this could be useful in promoting the therapeutic efficacy of BMSCs for clinical use.

#### Bone morphogenetic protein-2

Bone morphogenetic proteins (BMPs) belonging to the TGF-β family play roles in bone formation and cardiac diseases [88–90]. BMP-2 is a member of BMPs that has demonstrated therapeutic potential in MI by improving the contractility of cardiomyocytes and preventing cell death [91]. Whether it could regulate the cardiac differentiation of BMSCs requires further experiments. After treatment of BMSCs with BMP-2, Lv et al. [92] find that the differentiation of BMSCs into cardiomyocytes is enhanced by detecting the ultrastructural characterization, cardiomyocyte-specific protein expression, and mRNA expression of transcription factors. Furthermore, BMP-2 combined with Salvianolic acid B extracted from *Salvia miltiorrhiza* could produce better efficiency. To data, BMP-2 plays a role in inducing the cardiomyocyte differentiation of BMSCs and can play a synergy role with Salvianolic acid B [92].

BMP-4 could induce cardiomyogenic differentiation of human amniotic epithelial cells [93] and promote cardiac differentiation of mouse ES cells with autologous serum supplement [94]. BMP-10 comes to be critical in embryogenesis of the heart. As a member of the BMP family, BMP-2 is known to induce various types of stem cells into osteoblasts, chondrocytes, or adipocytes [95]. The BMP signaling pathway also plays key roles in regulating proliferation, differentiation, and survival of cardiac progenitor cells [96]. The expression of BMP-2 is increased after myocardial infarction, not only anti-apoptosis, but also regulating the cardiomyocyte differentiation of cardiac progenitors [97]. By controlling the expression of BMP-2, ES cells could differentiate into cardiomyocytes [98]. A previous study also shows that BMP-2 might differentiate BMSCs into a myocardial cell line. Salvianolic acid B could play a cardioprotective role in ES cell-derived cardiomyocytes in a hypoxia condition. Salvianolic acid B also could regulate the differentiation of various types of cells. For example, Salvianolic acid B promotes osteogenesis of human mesenchymal stem cells [99] and enhances BMSC differentiation into type I alveolar epithelial cells [100]. Salvianolie acid B could be used to induce myocardial differentiation of BMSCs due to its function of cardioprotective and regulationg differentiation.

## Microenvironment

Many research studies show that the cell-culture microenvironment may influence cell proliferation and differentiation. Recently, in-vitro studies have shown that culturing cells with specific medium could alter the cardiac-specific gene expression and differentiation of stem cells.

Wu et al. [101] utilize a high-voltage electrostatic field system to form nanosized collagen particles from collagen I solution. To further investigate whether collagen I nanomolecules could affect BMSC differentiation, BMSCs are cultured in medium with or without collagen I nanoparticles. After 24 h, 5-aza is added to induce the cardiomyocyte differentiation of BMSCs. The expression of two transcription factors (GATA4 and Nkx2.5) and four cardiac-specific markers (cTnI, β-MHC, CX43, and cardiac α-actin) are evaluated in BMSCs pretreatment with collagen I nanomolecules compared with BMSCs which not exposed to collagen I nanomolecules. These results demonstrate that collagen I nanomolecules can synergize with 5-aza to induce the cardiomyocyte differentiation of BMSCs, but the mechanism remains to be further explored.

Recently, in-vitro studies have shown that culturing substrates could modulate MSC differentiation [102]. Due to its physical and chemical properties and its effect on differentiation of MSCs [103], graphene has attracted much attention as a new type of MSC culture dish. To determine whether graphene could regulate the cardiomyocyte differentiation of human bone marrow-derived MSCs, Park et al. [104] conduct a series of studies. After cell seeding, cardiac-specific markers, including GATA4, cardiac actin, β-MHC, and cTnT, are all higher in MSCs cultured on graphene than in MSCs cultured on coverslips. Furthermore, the level of cardiomyogenic differentiation-associated extracellular matrix proteins (collagen I, collagen III, collagen IV, fibronectin, and laminin) in MSCs cultured with graphene supplement is increased. Taken together, these data suggest that graphene could promote cardiomyocyte differentiation of MSCs through differentiation-associated ECM proteins and related signaling pathways.

Collagen scaffold has been used as a cell product in clinical trials for cardiac repair [105]. A recent study shows that MSCs could enhance the expression of cardiomyocyte-specific proteins in collagen patches and secrete cardiotrophic factors [106]. Extracellular matrix is an essential property of the microenvironment cells interact with, and has a key role in influencing cell behavior and determining cell fate. Furthermore, MSCs cultured in collagen patches provide not only structural support to damaged myocardium but also promote tissue repair and enhance regenerative potential of MSCs [107–109]. Previous studies have shown that stem cell–extracellular matrix (ECM) interactions may take part in the cardiomyogenic differentiation of stem cells [110–112], whereas cardiomyogenic differentiation-associated ECM proteins can induce cardiac differentiation of ES cells [113].

Graphene-based materials have emerged with various functions in multiple biomedical applications, such as gene and drug delivery, cancer therapy, and tissue regeneration [114–116], due to their electrical and chemical properties. Moreover, they have been used to culture and differentiate stem cells [117, 118]. Ahadian et al. [119] find that graphene could induce spontaneous cardiac differentiation in embryoid bodies. Phosphorylated focal adhesion kinase (FAK) and ERK play an important role in regulating cardiomyogenic differentiation of stem cells [120]. Graphene could enhance stem cell adhesion, as the expression of FAK is increased [35], and it also increases the phosphorylation level of ERK. Park et al’s [104] results are also consistent with this. Western blot analysis results show that these differentiation-related pathways, FAK and ERK, are both activated in graphene-cultured MSCs.

## Others

### Caveolin-1

Caveolin-1 is an important part of caveolae, a specialized membrane invagination. Previous reports indicate that Caveolin-1 could play an important role in proliferation and differentiation of BMSCs [121–123], but the role of Caveolin-1 in cardiomyocyte differentiation of BMSCs remains unknown. Chen et al. [124] find that both mRNA and protein levels of Caveolin-1 are increased in 5-aza-treated BMSCs, suggesting that Caveolin-1 may be involved in the differentiation of BMSCs into cardiomyocytes. To further explore the role of Caveolin-1 in BMSC differentiation, Caveolin-1 siRNA is used. In the presence of siRNA, qRT-PCR and western blot analysis are performed to detect cardiac markers both at mRNA and protein levels, suggesting that knockdown of Caveolin-1 could enhance the cardiomyocyte differentiation of BMSCs [124].

The major finding of this study is that the downregulation of Caveolin-1 can promote the cardiomyocyte differentiation of BMSCs by regulating the activation of STAT3 signaling. Previous studies show that the expression of Caveolin-1 is increased in terminally differentiated mesenchymal lineage cells [125, 126], indicating that Caveolin-1 might prevent continued growth and differentiation. Guimaraes et al. [127] find that BMSCs isolated from the Caveolin-1 null mouse have an osteogenic differentiation potential, suggesting that Caveolin-1 could inhibit osteogenesis. Caveolin-1 also could block the neuronal differentiation and adipogenesis [128, 129]. The cardiomyocyte differentiation of BMSCs is a complex and multisignal process. As stated, STAT3 could enhance the differentiation of transplanted BMSCs, and inhibition of its activation could suppress the differentiation of mouse ES cells into cardiomyocytes induced by the cooperation of leukemia inhibitory factor and BMP-2 [130]. But this may be contradictory. Natarajan et al.’s [131] research shows that inhibition of STAT3 could promote neuron differentiation at the expense of astrogliogenesis. In this study, the activation of STAT3 is drastically decreased during inhibition of the expression of Caveolin-1 in BMSCs with or without 5-aza induction. Although the study demonstrates that Caveolin-1 plays an important role in cardiomyocyte differentiation of BMSCs through the STAT3 signaling pathway, the change of STAT3 activation is not consistent with previous reports and requires further investigation.

### Vanilloid receptor 1

Vanilloid receptor 1 (VR-1) is a Ca^2+^-permeable cationic channel belonging to the family of transient receptor potential ion channels [132]. The transient receptor potential ion channel family regulates neurons and osteocytes differentiation [133, 134]. VR-1 could promote osteoclast and osteoblast differentiation [135, 136]. Previous studies have indicated that VR-1 is expressed in cardiomyocytes and has roles in cardiac remodeling and differentiation from mouse ES cells to cardiomyocytes [137, 138]. Whether VR-1 could function in differentiation from BMSCs into cardiomyocytes is still not clear cut. After treatment with 5-aza, Ren et al. [139] find that the levels of VR-1 are evaluated in BMSCs, suggesting that VR-1 may play a role in the differentiation of BMSCs into cardiomyocytes. In order to further explore the potential role of VR-1 in cardiomyocyte differentiation of BMSCs, VR-1 is knocked down using siRNA. The mRNA and protein expressions of α-MHC, α-actin, and Nkx2.5 in the 5-aza group are significantly lower than those in the negative control group. These data show that VR-1 knockdown would inhibit the cardiomyocyte differentiation of BMSCs induced by 5-aza.

As previous evidence suggests that the Wnt/β-catenin signaling pathway is involved in myocardial development [140, 141], the authors postulate that the Wnt/β-catenin signaling pathway may be involved in the role of VR-1 in cardiomyocyte differentiation of BMSCs. However, subsequent experiments demonstrate that the expression of both β-catenin and ANIN2 positively correlated with the Wnt/β-catenin signaling pathway is reduced when VR-1 is knocked down in BMSCs. These results suggest that VR-1 could participate in the cardiomyocyte differentiation of BMSCs via the Wnt/β-catenin signaling pathway [139].

### Histone deacetylase 1

Currently, epigenetic modifications play an important role in cardiomyocyte differentiation processes of stem cells, especially histone acetylation [142, 143]. Previous studies have reported that HDAC1 knockdown could induce myocardial differentiation in P19CL6 cells and ES cells [144, 145]. In a preliminary experiment, the decrease of HDAC1 expression in BMSCs is identified during cardiomyocytes differentiation [146]. But whether HDAC1 is involved in the process is unclear. To further investigate this hypothesis, Lu et al. construct the optimal HDAC1-RNAi lentiviral vector to reduce the expression of HDAC1 in BMSCs. After treatment of BMSCs with the HDAC1-RNAi lentiviral vector, mRNA levels of cardiac-specific genes such as Nkx2.5, GATA-4, MHC, CX43, and cTnT are detected by qRT-PCR. The results show that the lentivirus-infected BMSC expression of these five genes is significantly higher than the vector or NC vector, indicating that inhibition of HDAC1 expression could promote the directional differentiation of BMSCs into cardiomyocytes [146].

Epigenetic modification of histone acetylation has been demonstrated to play significant roles in differentiation process of stem cells. Histone deacetylase (HDAC) has many subtypes that participate in this process, such as CUDC-907 regulating adipocytic differentiation of bone marrow stromal cells via HDAC [147], lncRNA H19 inhibiting adipocyte differentiation of BMSCs through HDAC4–6 [148], and HDAC8 suppressing osteogenic differentiation of bone marrow stromal cells [149]. On treatment of BMSCs with nonspecific inhibitors of HDAC such as suberoylanilide hydroxamic acid and trichostatin A which could suppress multiple HDAC subtypes except HDAC1, the expression of cardiomyocyte-specific genes is increased [142, 150]. In this study, cardiomyocytes differentiation is enhanced upon treatment of BMSCs with HDAC1-RNAi lentiviral vector, suggesting that HDAC1 also plays a role in cardiomyocytes differentiation of BMSCs.

## Prospects

In recent years, a series of research studies have provided different approaches to induce the cardiomyocytes differentiation of BMSCs, including chemical inducers, cytokines, microRNAs, culture intermediaries, and so on. Recent studies have also shown that hMSCs cocultured with primary cardiomyocytes could promote nuclear modification of hMSCs for cardiomyogenic-like cell differentiation [151]. But there are also series of problems, such as the carcinogenicity of 5-aza and the low differentiation efficiency of many inducers, all of which hinder the clinical application of BMSC transplantation. However, these research studies supply a train of thought for inducing BMSCs to differentiate into cardiomyocytes as well. Transplantation of BMSCs will become a new promising therapeutic strategy for clinical application in cardiac diseases in the future.

## Conclusions

**Fig. 1:**
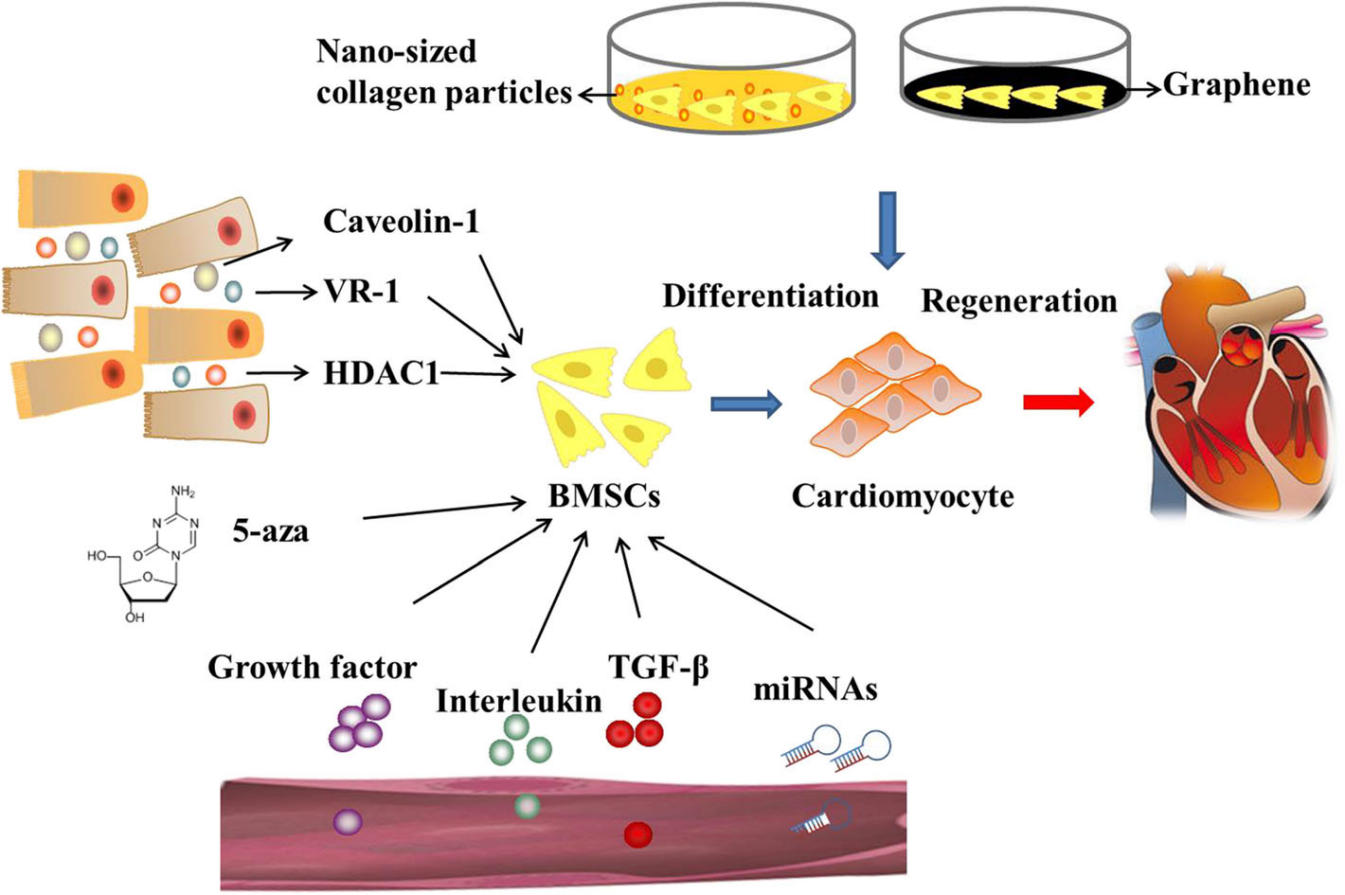
The regulators which can differentiate BMSCs into cardiomyocytes. BMSC bone marrow-derived mesenchymal stem cell, HDAC histone deacetylase, TGF-β transforming growth factor beta, VR-1 vanilloid receptor 1, 5-aza 5-azacytidine

Many researches have been done to explore the efficient therapy of cardiac diseases, and stem cells transplanting has been a promising therapeutic strategy. Stem cells could be induced into cardiomyocytes, and then migrated to damaged location to play the therapeutic effect. BMSCs would be the ideal source of stem cells due to easy availability, powerful capacity of proliferation and immune modulatory properties. In this article, some regulatory factors which could induce the cardiomyoctes differentiation have been summarized, including 5-aza, miRNAs, cytokines, microenvironment, Caveolin-1, VR-1 and HDAC1 (Fig. 1). More exploration are needed to elucidate the mechanism of BMSCs differentiate into cardiomyocytes and accelerate the clinical application.

## Abbreviations

AMI: Acute myocardial infarction
ANP: Atrial natriuretic peptide
5-aza: 5-Azacytidine
bFGF: Basic fibroblast growth factor
BMP: Bone morphogenetic protein
BMSC: Bone marrow-derived mesenchymal stem cell
CLC: Cardiomyocyte-like cell
cTnC: Cardiac troponin C
cTnI: Cardiac troponin I
cTnT: Cardiac troponin T
CX43: Connexin 43
Dll-1: Delta-like 1
ES: Embryonic stem
FAK: Focal adhesion kinase
GATA4: GATA binding protein 4
HGF: Hepatocyte growth factor
IGF-1: Insulin-like growth factor-1
iPS: Induced pluripotent stem
Islet-1: Insulin gene enhancer binding protein ISL-1
α-MHC: Alpha myosin heavy chain
Nkx2.5: Homeobox protein 2.5
pIGF-1R: Phosphorylation IGF-1 receptor
STAT3: Targeting signal transducers and activators of transcription 3
VR-1: Vanilloid receptor 1
